# Evolution and function of multimodal courtship displays

**DOI:** 10.1111/eth.12882

**Published:** 2019-05-10

**Authors:** Clémentine Mitoyen, Cliodhna Quigley, Leonida Fusani

**Affiliations:** ^1^ Department of Cognitive Biology University of Vienna Vienna Austria; ^2^ Konrad Lorenz Institute of Ethology University of Veterinary Medicine Vienna Austria

**Keywords:** elaborate displays, female preference, multisensory signals, sexual behaviour, sexual stimulation

## Abstract

Courtship displays are behaviours aimed to facilitate attraction and mating with the opposite sex and are very common across the animal kingdom. Most courtship displays are multimodal, meaning that they are composed of concomitant signals occurring in different sensory modalities. Although courtship often strongly influences reproductive success, the question of why and how males use multimodal courtship to increase their fitness has not yet received much attention. Very little is known about the role of different components of male courtship and their relative importance for females. Indeed, most of the work on courtship displays have focused on effects on female choice, often neglecting other possible roles. Additionally, a number of scientists have recently stressed the importance of considering the complexity of a display and the interactions between its different components in order to grasp all the information contained in those multimodal signals. Unfortunately, these methods have not yet been extensively adapted in courtship studies. The aim of this study was to review what is currently known about the functional significance of courtship displays, particularly about the role of multimodality in the courtship communication context. Emphasis is placed on those cases where a complete picture of the communication system can only be assessed by taking complexity and interaction between different modalities into account.

## 1 INTRODUCTION

Courtship is the suite of behaviours displayed by an individual to attract and eventually reproduce with an individual of the opposite sex (Bastock, 1967). Because courtship takes place within the scope of competition among conspecifics of the same sex, it is generally assumed to have evolved through sexual selection mechanisms. Courtship is usually performed by males towards females, but often involves an interaction between the two sexes (Huxley, 1914; Ota, Gahr, & Soma, 2015; Soma & Iwama, 2017), or a reversal of the usual sex roles. Historically, male courtship behaviour has been studied far more than female behaviour, and as a result, most of the examples we provide in this review refer to male courtship. However, our arguments apply equally well to female courtship.

Courtship displays are extremely diverse. They are known to occur in many sensory modalities and can vary substantially even between closely related species (Andersson, 1994; Bastock, 1967). The most studied courtship displays in the animal kingdom are the visually conspicuous dances and acoustic calls of birds. Vibratory and olfactory signals are also very common, especially in arthropods (Hebets & Uetz, 1999; Houck & Reagan, 1990). Courtship can vary in its duration, with some species having only a few seconds courtship interaction before mating (Bastock & Manning, 1955), to several days of mutual interaction before copulation, as in the dwarf seahorse (*Hippocampus zosterae*) (Masonjones & Lewis, 1996) or emperor penguin (*Aptenodytes forsteri*)(Ancel, Gilbert, & Beaulieu, 2013). Secondary sexual characters and courtship are generally more conspicuous and intense in polygamous species, but are also present in socially monogamous species (Kirkpatrick, Price, & Arnold, 1990).

Past work focused mainly on the most conspicuous component of courtship signals (typically visual or auditory). However, with the possible exception of courtship occurring in environments in which some modalities cannot be transmitted, such as in complete darkness, most courtship displays occur in at least two sensory modalities and include more than one signal aimed at separate sensory systems of the receiver. Those components may occur sequentially at different times during courtship. This is the case when males use the first component to attract females from a distance, and then another when the female is at a closer range, for example in the ring‐necked pheasant *Phasianus colchicus* (Mateos & Carranza, 1999). Alternatively, the components can occur simultaneously, leading to very complex signals such as in birds performing conspicuous courtship composed of dances and calls (Andersson, 1994). We refer to these types of courtship as multicomponent if the components occur in the same sensory modalities, and multimodal or multisensory if they occur in two or more sensory modalities (Partan & Marler, 2005; Rowe, 1999). Complex multicomponent and multimodal courtship displays have now been described for a large number of taxa (Knörnschild, Feifel, & Kalko, 2014; Manica, Macedo, Graves, & Podos, 2017; Mowles, Jennions, & Backwell, 2017; Ota et al., 2015; Preininger et al., 2013), and for both sexes. For example, in the blue‐capped cordon‐bleu (*Uraeginthus cyanocephalus*), Ota et al. (2015) documented a multimodal courtship display composed of visual, auditory and tactile components performed by both males and females during courtship interactions.

Courtship plays an essential role in reproduction as it is often required for copulation to occur, with the exception of sneaky and forced copulations. Yet the ultimate causes at work are still poorly understood, as the links between the multiple signals of complex, elaborate displays and fitness benefits are far from being clear. In particular, in the context of multicomponent courtship displays, it is still unclear what the role of each component is and why very elaborate behaviours have sometimes evolved in cases where simpler displays should suffice. As Candolin (2003) noted, courtship behaviour is usually studied as a simple structure, even though it almost invariably includes several components. Hundreds of studies have focused on single components of male courtship and investigated their relevance for female choice or female stimulation. With surprisingly few exceptions, studies on multimodal courtship rarely examine how females integrate multiple components to reach mating decisions, or whether courtship signals have effects beyond mate choice. The traditional “trait‐based” approach assesses the effects of individual signals sent by males on female preference and choice (Schacht & Grote, 2015). Experimentally, this is achieved via cue‐isolation experiments, where single stimuli are presented separately to the choosing sex, typically the female. This approach raises at least three issues. First, experiments of this type rarely accurately reflect the complexity of courtship interactions as they occur in nature. Even in cases in which single modality components may reach the receiver in isolation from the rest of the signal, this does not always occur in a symmetric way in the wild. For example, in audiovisual communication, the sender could be visually hidden but easily heard, whereas the opposite scenario is unlikely unless there is masking by strong background noise. Cue‐isolation experiments have rarely taken this into account and usually involve playback stimuli separated from other stimuli that typically would accompany them. Such experiments therefore often disregard the natural conditions in which the behaviour evolved. Second, by focusing on the role of courtship on female choice and preference, researchers often neglect other potential functions of courtship. For instance, few studies have examined the role of male courtship components on female sexual stimulation, which may have a strong influence on male reproductive success (Beach, 1975; Lehrman & Friedman, 1969). Finally, the trait‐based approach ignores information contained in the interaction between different components of courtship signals. An increasing number of theoretical (Candolin, 2003; Hebets & Papaj, 2005; Partan & Marler, 1999; Stein & Standford, 2008) and empirical studies (Ronald, Zeng, White, Fernandez‐Juricic, & Lucas, 2017; Stange, Page, Ryan, & Taylor, 2016; Taylor & Ryan, 2013) have shown that the response to multiple signals differs from the simple sum of the responses to each component taken separately. This emphasizes the potential additional value provided by the interaction between components (Taylor & Ryan, 2013).

Our overall goal is to provide a concise overview of what is known today about multimodal courtship displays, from both the empirical and theoretical point of view. For recent reviews about more general themes linked to the present paper, see Rosenthal (2017) for a thorough review of mate choice, Prum (2012) for a novel perspective on the role of beauty and attractiveness in sexual selection, and Ryan (2018) for a complete overview of the sensory bias theory. Because identifying the different functions of a particular behaviour is fundamental to understanding its evolution, the first aim of this paper was to review what is currently known about the general roles of courtship displays. Then, we will discuss how different signals can be composed to form complex multicomponent or multimodal courtship displays. Finally, with the help of recent theoretical work regarding multimodal communication, we will focus on assessing the function of complex behavioural signalling in the reproductive context to stress the importance of studying such signals as a complex unity.

## 2 FUNCTIONAL SIGNIFICANCE OF COURTSHIP

The most studied function of courtship is that of highlighting male quality and thus increasing reproductive success by enhancing the chances of the male to obtain a mate. However, courtship displays have other important functions.

### 2.1 Sex and species recognition

The ability of an organism to recognize members of its own species, and in particular of the opposite sex, is fundamental to produce offspring. In various taxa, closely related species might be morphologically very similar. In such cases, courtship behaviour can serve to identify members of a particular species, thereby reducing the risk of inter‐specific mating in sympatric species. In the *Drosophila* genus, several features of courtship songs vary between species and are thought to be responsible for maintaining sexual isolation between closely related species (Saarikettu, Liimatainen, & Hoikkala, 2005). Another example comes from a recent study on birds of paradise (*Lophorina* genus), where the authors analysed the audiovisual courtship displays of individuals in New Guinea (Scholes & Laman, 2018). By highlighting differences in ornament exposition in the courtship displays of birds previously thought to belong to only one species, they could support previous molecular and morphological analysis (Irestedt, Batalha‐Filho, Ericson, Christidis, & Schodde, 2017) and confirm the existence of several allopatric species. As a further example, in field crickets of the *Teleogryllus* genus, male calls that serve to attract females are thought to be important for pre‐zygotic isolation (Hoy, Hahn, & Paul, 1977). Finally, in *Heliconus* butterflies, male multimodal courtship based on olfactory and visual signals is thought to be a powerful driver of reproductive isolation (Southcott & Kronforst, 2017). Similarly, in monomorphic species where the two sexes look alike, courtship and response to courtship provide important information about the sex of the potential partner, for example in the ring dove (Lehrman, 1964; Lovari & Hutchison, 1975). Even though reproductive isolation due to mate choice has been long seen as a main driving force of speciation (Lande & Kirkpatrik, 1988), in some cases, courtship might not be sufficient to maintain reproductive isolation. In Drosophila heteroneura and Drosophila silvestris, for example, experimental crosses between the two species showed that courtship behaviour has a minor role in reproductive isolation (Boakes, DeAngelis, & Andreadis, 1997) and that the isolation between the species is due to the failure of heterospecifics to perform courtship behaviour at all, rather than differences in courtship repertoire such as courtship duration (Boakes, Andreadis, & Witzel, 2000).

### 2.2 Sexual stimulation and synchronization of mating behaviour

In species with a distinct breeding season, the transition to reproductive status is triggered by environmental factors such as photoperiod, temperature or light intensity (Farner, 1964; Gemeno & Haynes, 2001). In some species, additional stimulation is sometimes necessary for mating to occur, and courtship and mating can induce the female to become physiologically responsive and eventually allow fecundation.

Within this context, a considerable amount of research has been carried out on web‐building spiders. In orb‐web spiders, one function of the abdominal wagging performed by the courting male on the female is thought to be an increase in pressure of the haemolymph which facilitates sperm transfer after copulation (Huber, 2004; Wignall & Herberstein, 2013). In the wolf spider (*Stegodyphus lineatus)*, courtship pre‐mating vibratory behaviour seems to stimulate the receptive female to mate (Maklakov, Bilde, & Lubin, 2003). In salamander of the Plethodontidae family, sexual pheromones delivered by males during courtship shorten the latency of females to mate and increase female sexual receptivity (Houck & Reagan, 1990; Houck et al., 2008). In the ring dove (*Streptopelia risoria*), male courtship is responsible for hormonal and physiological changes that trigger oviduct growth in females, making reproduction possible (Lehrman, 1964). Lovvorn, Mossotti, Wilson, and McKay (2012) described the courtship behaviour of spectacled eiders (*Somateria fischeri*) and hypothesized that the more likely role of courtship in this species was to accelerate female hormonal development in order for them to be ready to mate during the short time windows their polar habitat offers.

Courtship can also be useful for spatial synchronization of mating behaviour in species where individuals are spatially dispersed, for example as a means to attract females to a courting male's territory or to a breeding site. In field crickets, males use a long‐range calling song to attract distant females (Alexander, 1961). The use of long‐distance infrasound calls has also been proposed to attract females to mating leks in cetacean species where individuals can be several hundreds of kilometres apart (Herman, 2016).

### 2.3 Female choice process

According to sexual selection theory, females choose a sexual partner because of the relatively greater benefits potentially acquired through mating with this individual. Those benefits are traditionally classified as direct if females gain access to territory, help in parental care or other resources, and indirect if they are gained only by the offspring, for example, good genes and/or the capacity to attract mates (Andersson, 1994). With courtship, males may signal potential benefits to females, and females can assess these signals to make a mating decision. A large number of male morphological traits have been shown to play a role in the female choice process, among them size (Harari, Handler, & Landolt, 1999), symmetry (Little, Jones, DeBruine, & Feinberg, 2008) and colour (Kodric‐Brown & Nicoletto, 2001). Some studies examined the link between particular features of courtship and female preference and choice. Among the features under female selection, we find rate and intensity of postural (Mowles, Jennions, & Backwell, 2018) or auditory (McComb, 1991) displays, as well as total courtship duration (Seymour & Sozou, 2009) and overall courtship rate (Berson & Simmons, 2018). In the golden‐collared manakin (*Manacus vitellinus*), females show a preference for good motor skills by choosing to mate with males who display faster and longer (Barske, Schlinger, Wikelski, & Fusani, 2011; Fusani & Schlinger, 2012). All those characters are thought to indicate physical ability or general qualities of the males that can be passed to the offspring.

Not all male characters necessarily reflect some intrinsic quality. In some species, males are known for exploiting female sensory biases to influence their decisions. Sensory bias theory states that the most successful courtship displays are those which best stimulate specific aspects of the female sensory system which evolved through natural selection (Fuller, Houle, & Travis, 2005; Rowe, 1999). Although the sensory bias hypothesis has been applied to specific morphological and acoustic traits involved in mate choice (see reviews by Ryan & Cummings, 2013), examples of its extension to more elaborate courtship displays are rather scarce. One example comes from a study on grasshoppers (*Chorthippus biguttulus)* where the authors studied the shape of the female preference function after artificially adding a new element to a naturally simple male courtship and thereby making it more complex (Reichert, Finck, & Ronacher, 2017). They found a complex relationship between female preference and the timing and the type of novel elements added to the original song and concluded that sensory bias could in some cases promote the evolution of male courtship signals. Another example comes from different species of bowerbirds, where it has been found that male preference for the coloured decorations they use to adorn their bowers matches with female colour preferences for food items during foraging (Madden & Tanner, 2003). A more sophisticated case of exploitation of a courtship receiver's perception has been suggested in the case of the great bowerbird (*Ptilonorhynchus nuchalis*), where males place objects in a particular size order when building the court in front of their display avenue, creating a visual illusion which might make the displaying male look larger than he really is (Kelley & Endler, 2017).

During the early discussions about mate choice within an evolutionary context, Wallace argued that courtship vigour was the primary focus of females, while Darwin thought that the choice for ornamentation prevailed (Hoquet & Levandowsky, 2015; Prum, 2012). Both of them were probably right, as ornaments and vigour are often closely related (Cornuau, Rat, Schmeller, & Loyau, 2012), as motor displays or specific postures are necessary to expose ornaments and to make them more conspicuous (Hebets & Uetz, 2000; Jones, Byrne, & Wallman, 2014).

### 2.4 Moderation of female aggression

Additionally, courtship may act as a moderator of female aggressiveness and is particularly important in species where female cannibalism is common. In these cases, males should greatly benefit from displaying a behaviour that may prevent them from being killed. In orb‐web spiders (*Argiope keyserlingi)* for example, male shuddering behaviour during courtship seems to have an effect on female cannibalism (Wignall & Herberstein, 2013).

## 3 WHY SO MUCH COMPLEXITY IN COURTSHIP DISPLAYS?

The presence of complex communication signals raises questions about their advantage over simpler ones. Producing complex signals might be energetically more costly and might increase predation rate (Partan & Marler, 2005) (but see Clark, 2012 for an alternative view on the potential cost of courtship, and Munoz and Blumstein (2012) regarding the cost of multisensory signals in general). Although it is clear in some cases that multimodal signalling improves mating success (Berson & Simmons, 2018; Girard, Elias, & Kasumovic, 2015; Stafstrom & Hebets, 2013) or increases physiological responses in females (Friedman, 1977), the proximate and ultimate mechanisms involved are unclear and elaborate behavioural signalling still lacks a unitary and broadly accepted theoretical framework.

The evolution of multicomponent signalling has recently received a great deal of attention, and several hypotheses have been proposed (Partan, 2013). For example, Hebets and Papaj (2005) stressed the fact that selection pressure can also act on the composed signal and not only on its independent components. Following the classification first proposed by Guilford and Dawkins (1991), the authors distinguish between “content‐based” and “efficacy‐based” hypotheses as possible mechanisms of complex signalling evolution. While the former focuses on the information carried by the signal, typically identity or quality in the context of courtship displays, the latter includes mechanisms improving the production, transmission and reception of a signal, including factors in the signalling environment or the receiver's sensory system. Later, Rowe and Halpin (2013) applied this same classification to the specific case of aposematic signals. Candolin (2003) conceived a similar classification for cues used in mate choice in a large number of taxa, but did not specifically address courtship or courtship components other than those involved in mate choice. Table 1 lists empirical studies reporting evidence for the benefits of multimodal courtship displays.

**Table 1:** Empirical studies reporting a benefit for sender and/or receiver for courtship composed of more than one sensory modality

Function of multicomponent/multimodal signals	Sensory modalities involved	Species	References
**Improve signal efficiency**			
Vocal sac helps females to better discriminate and detect male signal	Visual and acoustic	Anurans *sp*	Starnberger et al. (2014)
Part of the auditory courtship increases discriminability of the entire call	Acoustic	Magicicada *sp*	Cooley and Marshall (2001)
Redundancy, that is suppression of one modality does not alter copulation success	Visual, acoustic, chemical and tactile	Drosophila (*Drosophila saltans*)	Colyott et al. (2016)
**Provide multiple information about male qualities**			
Vibration vigour and display duration advertise a different aspect of male quality and differentially predict mating success	Visual and tactile	Peacock spider (*Maratus volans*)	Girard et al. (2015)
Different male display traits predict different cognitive abilities of the males	Visual	Satin bowerbird (*Ptilonorhynchus violaceus*)	Keagy et al. (2012)
**Trigger different females responses**			
Pheromones serve for sex recognition and head bobbing attracts the attention of females and communicates male's location	Chemical and visual	Iguana (*Liolaemus pacho)*	Vicente and Halloy (2016); Vicente and Halloy (2017)
Frequency and temporal patterns of sounds give information about species identity while call intensity and visual signals influence mate choice	Acoustic and visual	Sand goby (*Pomatoschistus minutus*)	Pedroso et al. (2013)
Shuddering behaviour increases female acceptance and reduces aggressiveness while abdominal wagging facilitates sperm transfer	Visual and tactile	Orb‐web spider (*Argiope keyserlingi*)	Wignall and Herberstein (2013); Huber (2004)
**Reach different receivers**			
Females differ in their preference for individual components of courtship according to their own sensory configuration	Visual and acoustic	Brown‐headed cowbird (*Molothrus ater*)	Ronald et al. (2018)
Acoustic part help males in discriminating between female visual aggressive and courtship display	Visual and acoustic	Red‐winged blackbirds (*Agelaius pboeniceus*)	Beletsky (1983)
**Signal at different environmental scales**			
Feeding courtship attracts female attention and the lateral display triggers copulation solicitation displays by females	Visual	Ring‐necked pheasant (*Phasianus colchicus*)	Mateos and Carranza (1999)
Acoustic component determines whether females visit a male and display rate and then predicts the likelihood of mating	Visual and acoustic	Sage grouse (*Centrocercus urophasianus*)	Gibson (1996)
**Signal good neuro‐muscular coordination**			
Courtship elicit female response only if all the components are present	Visual and tactile	Drosophila (*Drosophila virilis*)	LaRue et al. (2015)
Males synchronize acoustic with visual components	Visual and acoustic	Montezuma oropendolas (*Psarocolius montezuma*)	Miles and Fuxjager (2018a, 2018b)
Temporal synchrony of signals increases female receptivity	Visual and tactile	Brush‐legged wolf spider (*Schizocosa ocreata*)	Kozak and Uetz, (2016)
Females reject courtship when the signal lacks synchrony and synchronized signals are more attractive	Visual and acoustic	Túngara frogs (*Physalaemus pustulosus)*	Taylor et al. (2011); Taylor et al. (2017)
**Interaction between components yields new information**			
Two artificial courtship signals individually not attractive combine into an artificially attractive signal	Visual and acoustic	Túngara frogs (*Physalaemus pustulosus)*	Taylor and Ryan (2013)
Female integration of component signals is not additive, and the preference varies with signal complexity	Visual and acoustic	Túngara frogs (*Physalaemus pustulosus)*	Stange et al. (2016)
Intensity of the visual component of male courtship modulates the attractiveness of male song	Visual and acoustic	Brown‐headed cowbird (*Molothrus ater)*	Ronald et al. (2017)

### 3.1 Improving signal efficiency

Rather than carrying information for the receiver, some parts of a multicomponent signal can instead act to improve signal efficiency, which is defined as the probability that the receiver perceives the signal in an intended way. This can be achieved by improving the way the receiver perceives the signal (receiver psychology hypothesis), or by improving transmission in the environment (backup signals hypothesis).

#### 3.1.1 Receiver psychology in the courtship context

The receiver psychology hypothesis states that some signal components function to facilitate improved perception, discriminability, assessment or memorization of the information contained in the main signal (Guilford & Dawkins, 1991; Rowe, 1999). For instance, a sound can function to draw attention to a visual display, or vice versa. In anurans, for example, vocal sac inflation during courtship calling helps females to detect and discriminate male signals, thereby increasing male attractiveness (Starnberger, Preininger, & Hödl, 2014). Receiver psychology in the courting context is particularly relevant in noisy environments where the assessment of mates is difficult. For example, in a study on sexual signalling in several *Magicicada* species, Cooley and Marshall (2001) hypothesized that some parameters of the auditory courtship display increase the discriminability of individual calls among a chorus. It has long been established that low‐intensity signals are detected faster when they occur in more than one modality (e.g., Gielen, Schmidt, & Heuvel, 1983). This seems to equally apply to multicomponent and multimodal signals. Indeed, in an experiment with swordtails (*Xiphophorus nigrensis*), it was found that females were faster to approach one of two males when the males differed on two rather than only one visual signal (body size and courtship vigour) (Reding & Cummings, 2017).

#### 3.1.2 Back‐up signals hypothesis

The back‐up signal hypothesis specifies that multicomponent signals carry redundant information to limit errors in signalling, allowing the receiver to assess the final message with more accuracy (Johnstone, 1996; Møller & Pomiankowski, 1993). In those cases, we expect different traits to be correlated as the multiple “back‐up” components are redundant if the receiver's response to each is the same. Bro‐Jørgensen (2010) hypothesized that even though one signal might be sufficient to communicate a message, temporal and/or spatial environmental variability could lead to the evolution of multicomponent sexually selected signals (“fluctuating environment hypothesis,” Munoz and Blumstein (2012) and Partan (2017)). Multicomponent displays could prevent interference from unpredictable variation and thereby ensure signal transmission under varying environmental conditions. In such cases, it is predicted that back‐up signals would evolve. For example, Colyott, Odu, and Gleason (2016) found that removing one courtship component (it being either visual, auditory, chemical or tactile) in *Drosophila saltans* did not alter the females’ decision to mate. This indicates at least some degree of redundancy between different components. In the satin bowerbird (*Ptilonorhynchus violaceus*), Keagy, Savard, and Borgia (2012) found that females used multiple traits of the bower constructed by the male, such as the size of the sticks used, the symmetry of the bower, or the colouration of decorations, to better estimate a composite measure of male cognitive abilities. They additionally found that some of those traits were redundant, for example stick size and bower symmetry seem to convey the same information. In canaries (*Serinus canaria*), female responses to male courtship are multimodal and composed of a visual (copulation solicitation display, CSD) and auditory part (female‐specific trill, FST and contact calls, CC) (Amy, Salvin, Naguib, & Leboucher, 2015). Salvin (2018) found that the number of modalities used by both males and females within courtship interactions affected the response of the other sex. For example, males responded to a female's behaviour during courtship only when they could see and hear the female and not when they could only hear them. However, no enhancement effect between female visual and auditory signals seemed to occur, and those two signals seemed to be redundant for males.

### 3.2 Multicomponent courtship for multiple pieces of information

In contrast to the above hypotheses, the multiple message hypothesis states that each component of the multimodal signal carries different information, and therefore, each component taken separately should trigger a different response (Johnstone, 1996; Møller & Pomiankowski, 1993). For example, theoretical work by Wilson, Dean, and Higham (2013) investigated which constraints would favour the evolution of multimodal signals over simpler signals and found that having multiple receivers or multiple qualities to display would all favour the emergence of multimodal signalling.

Most of the studies on multicomponent signals in the context of courtship displays focus on behavioural responses indicating female preference (Ronald et al., 2017; Taylor & Ryan, 2013), but less has been done on multicomponent courtship where separate components might have different functions (such as sexual stimulation of the female, cf. part 1 above).

#### 3.2.1 Signalling different aspects of male quality

According to sexual selection theory, females choose a male who can increase their reproductive success or the quality of her offspring. In an interesting theoretical paper, van Doorn and Weissing (2004) showed that the evolution of multiple male ornaments was plausible if they displayed different aspects of male quality. Although they focussed on ornaments, the same evolutionary process could be involved in the behavioural components of courtship.

Surprisingly, very few studies have focused on the multiple aspects of male quality that courtship could potentially advertise. In some cases, multimodal signals have been found to display different aspects of a male's quality (multiple message hypothesis). Girard et al. (2015) found that in peacock spiders (*Maratus volans),* different components of courtship, such as vibration vigour and display duration, advertise separate aspects of male quality and differentially predicted mating success. Another example comes from the satin bowerbird, where features of the bower and plumage related to courtship were found to reflect different aspects of male quality (Doucet & Montgomerie, 2003).

#### 3.2.2 Triggering different female responses

There is also evidence that different parts of the male display can trigger differential responses in females. In the iguana *Liolaemus pacho* for example, chemical signals are thought to have a role in sex recognition (Vicente & Halloy, 2016), while other behaviours like head‐bobbing might have a role in attracting the attention of the receiver and communicating the signaller's location (Vicente & Halloy, 2017). In the sand goby (*Pomatoschistus minutus*), the frequency and temporal pattern of sounds are thought to communicate species identity, whereas call intensity and the visual part of male courtship are more relevant for female choice (Pedroso, Barber, Svensson, Fonseca, & Amorim, 2013). In the orb‐web spider, as mentioned above, some of the courtship signals have an influence on female acceptance and probably reduce female aggressiveness (Wignall & Herberstein, 2013), while other signals might facilitate sperm transfer (Huber, 2004). In the lizard *Anolis carolinensis*, early investigations by Crews (1975) on the relative importance of each component of the male courtship display showed that physical movements of the male dewlap during courtship triggered hormonal changes and follicular growth in females, while dewlap colour was used by females for mate choice.

#### 3.2.3 Reaching different receivers

In some cases, multimodal signalling can help the signaller to reach more than one receiver. Female preference has long been considered to be homogeneous within a studied population, as if a consensus had been reached about attractiveness of male attributes. A commonly used approach is therefore to use the mean female response of the population as the response variable. However, it is becoming more and more evident that mate preference varies greatly depending on a female's age, condition or environment (Burley & Foster, 2006; Jennions & Petrie, 1997). Female preference for male colouration can vary according to the level of predation risk (Godin & Briggs, 1996). It is thus likely that female preference for individual courtship components varies within a population. Displaying on different sensory channels, and having more than one information‐containing signal, would potentially allow a male to attract different types of females, thereby dealing with variation in female preferences. In the brown‐headed cowbird (*Molothrus ater*), females differ in their preferences for individual components of the complex male courtship display as a function of their sensory acuity (Ronald, Fernández‐Juricic, & Lucas, 2018). In this species, the visual and auditory temporal resolution of females has an influence on which type of visual display and songs they prefer during male courtship, for example females with better auditory temporal resolution preferred shorter songs.

In other cases, some components might be relevant not only in the context of courtship, but also function as signals directed to other males or predators. In the flamboyant lizard (*Sarada superba)*, different colours of the same display elicit different responses in rival males and courted females, stressing the role of simultaneous selection pressures from intra‐ and inter‐sexual selection in the evolution of multimodal signals (Zambre & Thaker, 2017). In several bird of paradise species, display complexity is also driven by those two forces (Miles & Fuxjager, 2018a,2018b). Female choice seems to influence sexual dichromatism, while male–male competition is related to carotenoid‐based ornaments. In the peacock (*Pavo cristatus*), different signalling ornaments and behaviour have evolved in response to pressure coming from both intra‐ and inter‐sexual selection (Loyau, Saint Jalme, & Sorci, 2005) and in the ochre‐bellied flycatcher (*Mionectes oleaginous)*, male songs in leks are used both for courtship to females and intra‐sexual competition (Westcott, 1992). The case of co‐option of courtship behaviour from aggressive displays is also quite common in the animal kingdom (Berglund, Bisazza, & Pilastro, 1996). In the ring dove, for example, the bow‐coo display used as an aggressive display towards other males does not differ from the courting display males perform to attract females (Craig, 1909). When the courtship display and the aggressive display are similar, an additional signal component may act as an indicator of intention (Baptista, 1978). For example, in female red‐winged blackbirds (*Agelaius pboeniceus*), the visual signals used for courtship and aggressive display by territorial females are similar, while two types of female song have been reported. This suggests that the auditory part of the signal allows a male to disambiguate the female's behaviour (Baptista, 1978; Beletsky, 1983).

#### 3.2.4 Signalling at different geographical and temporal scales

Sometimes, having several courtship components can reduce the cost of mate choice by reducing the time females spend in close inspection of available males. For example, females can use one signal to choose which males are worth observing and then use another signal component for their subsequent choice among this subset of males. In other words, different cues are used for attraction and for mate assessment. Although the sequential assessment of cues to gain information from a conspecific is well documented in general (Uy & Safran, 2013), less evidence exists in the context of mate choice, and even less in the general context of courtship. In the fiddler crab for example, females first select certain males for their size and then assess them for their burrow quality in order to decide which male to mate with (Backwell & Passmore, 1996).

Regarding the specific case of courtship, in the ring‐necked pheasant (*Phasianus colchicus*), different messages sent at separate times by males during courtship elicit different responses in females (Mateos & Carranza, 1999). The first signals are used to attract females’ attention, whereas the “lateral” display triggers a copulation solicitation display from the female.

At a larger scale, some elements of courtship can attract the potential mate and guide her towards the sender. In the sage grouse (*Centrocercus urophasianus)*, females use the acoustic component of the display to determine whether to visit a male, but the display rate is a better indicator of the likelihood of mating (Gibson, 1996).

### 3.3 Information in complex signal structure and component interactions

Partan and Marler (1999) were the first to propose a classification of multimodal signals in animal communication. They pointed out that adding a second component to a unimodal signal could modulate the first component's effects on the receiver's behaviour (e.g., enhancement or suppression), or even create a new “emergent” response in the receiver. Their article was an important contribution to multisensory communication theory in the field of animal behaviour, because historically multicomponent signals were studied by analysing each component separately and not by taking the whole signal or the relationship between components into account. More recently, an increasing number of authors have emphasized the need for studying complexity itself, as the different components of a signal are likely to have evolved conjointly (Cooper & Goller, 2004; Groyecka et al., 2017).

**Figure 1:**
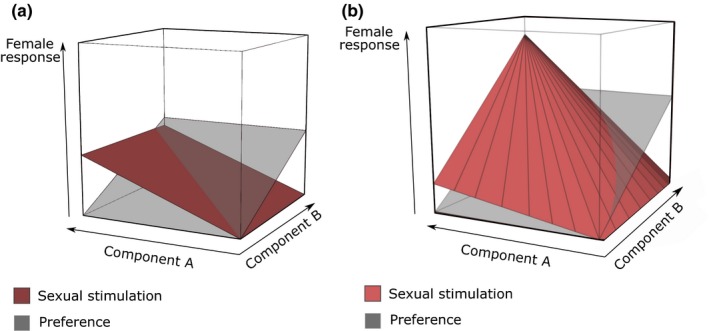
Multidimensional plots of female responses to multicomponent male courtship. Components A and B have different effects on female preference and female sexual stimulation, resulting in two three‐dimensional planes. (a) No interaction between components A and B. As component A increases, female sexual stimulation increases, but not female preference. Conversely, component B has a positive effect on female preference but does not influence female sexual stimulation. (b) An interaction exists between courtship components A and B. The effect of component A on female sexual stimulation increases as the magnitude of B increases. Component B alone does not have any effect on female sexual stimulation. [Based on Hebets et al. (2016) and Smith and Evans (2013)] [Colour figure can be viewed at wileyonlinelibrary.com]

Smith and Evans (2013) described an interesting heuristic for the study of multicomponent signals. In particular, they proposed a method to better understand and visualize how concomitant variation within and between two modalities influences the receiver's response. The resulting three‐dimensional surface plot (with the magnitude of each component signal represented on two axes and magnitude of female response on the third) was later used by Hebets et al. (2016) to study female preference in response to two courtship stimuli, using the multisensory wolf spider courtship display as a model. This type of graphical representation helps to visualize the complexity of a receiver response when exposed to different levels of components inside the same complex display. Here, we use a similar graphical representation to describe how two male courtship components can separately or jointly influence two female responses (Figure 1). The two‐plane multidimensional plots illustrate a theoretical case where two sensory modalities of male courtship (e.g., a visual and an auditory signal) can interact or not to influence two aspects of female response (e.g., female preference and female sexual stimulation).

One of the first examples of a behavioural response that derives specifically from a multisensory display comes from the study from Rowe and Guilford (1996) who investigated multicomponent anti‐predator warning signals found in prey. They found that neither the chemical released by the prey nor the warning colour they display could, when presented alone, trigger aversive behaviour in the domestic chick (*Gallus gallus domesticus*) and that the repulsive effect emerged only when the two signals (visual and chemical) were combined. This stresses the importance of taking each component into account, as well as paying attention to the overall structure of the complex signal and the relation between its different components.

In the specific case of courtship, most of the studies reporting an interaction between signals do not directly concern interactions between different behavioural components of courtship, but rather the relative role of courtship behaviour and morphological traits (Reynolds, Gross, & Coombs, 1993). See Table 2 for an overview of studies investigating multiple courtship components.

**Table 2:** Definitions of key terms employed in this review

Term	Definition	Example	References
Multicomponent courtship	Courtship with two or more distinct signals occurring in a single sensory modality	The vocal courtship of the túngara frog composed of whines and chucks	Partan and Marler (2005); Rowe (1999); Stange et al. (2016)
Multimodal/multisensory courtship	Courtship comprising two or more signals occurring in at least two different sensory modalities	The bowing display of a ring dove, including a visual signal (the bow) and the bow call	Partan and Marler (2005); Rowe (1999); Fusani, Hutchison, and Hutchison (1997)
Complex courtship	Courtship composed of multiple signalling elements, in one or more sensory modalities	The acrobatic dance of a manakin, jumping in a courtship arena while displaying brilliant plumage and snapping its wings	Fusani, Giordano, Day, and Schlinger (2007); Hebets and Papaj (2005);Miles and Fuxjager (2018a,2018b); Perrot et al. (2016)
Female sexual stimulation	Physiological changes occurring in females following male courtship	Courtship‐induced follicular growth in ring doves	Friedman (1977)

#### 3.3.1 Multisignalling as a marker of quality per se

Independently of its content, multicomponent signalling can intrinsically be a sign of the quality of the displaying individual. Indeed, complex courtship displays are often more energetically costly than single‐component displays and could therefore be under female selection for energetically demanding behaviour (Byers, Hebets, & Podos, 2010). For example, in *Drosophilia virilis,* courtship elicits a response from a female only if all elements of a sequence of signals are present. This might be a way for females to select those males that are able to send accurately timed and energetically costly signals (LaRue, Clemens, Berman, & Murthy, 2015). In the wolf spider (*Schizocosa ocreata*), females prefer multimodal courtship signals over unimodal ones (Stoffer & Uetz, 2017). Additionally, in peacock spiders (*Maratus volans*), Girard et al. (2015) found that total courtship effort (a variable capturing the time a male spends courting across different sensory modalities) positively affected female preference. We know that in some species, courtship is costly and condition dependent. In the fiddler crab (*Austruca lactea*) for example, males produce a multisensory courtship display to attract females from a distance and then to court them when closer. Takeshita, Murai, Matsumasa, and Henmi (2018) showed that the male visual waving signal used to attract females was condition dependent and therefore could be used by females to assess the individual quality of potential partners.

In addition, the timing of different components of courtship does not occur randomly. Signal synchronization could itself indicate good neural control. In the Montezuma oropendolas (*Psarocolius montezuma*), males synchronize the loudest note of their song with a specific visual courtship display (the bow and wing‐spread), which could indicate the quality of an individual motor's skills (Miles & Fuxjager, 2018a,2018b). In the brush‐legged wolf spider (*Schizocosa ocreata*), accuracy in the temporal synchrony of separate courtship signals increases female receptivity (Kozak & Uetz, 2016). In Túngara frogs (*Physalaemus pustulosus)*, females reject the courtship when the multimodal elements of the mating signal lack synchrony (Taylor, Klein, Stein, & Ryan, 2011). In the same species, synchronized visual and acoustic displays are more attractive to females than asynchronous signals (Taylor, Page, Klein, Ryan, & Hunter, 2017). However, an asynchronous multimodal signal is still more attractive than a unimodal signal. This underlines the complex relationship that can exist between all sensory modalities contributing to a signal and the information contained in their interactions and their relative timing.

#### 3.3.2 Multimodal courtship displays are more than the sum of their parts

When we consider the response to a complex signal that involves several sensory modalities, we now realize that this response is not always additive. Experiments investigating multisensory integration in the midbrain have found clear evidence for super‐additive multisensory enhancement when comparing single neurons’ responses to uni‐ and multimodal stimuli (Meredith & Stein, 1983). Multisensory responses have also been found in cells located in cortical areas of the rodent brain which were traditionally assumed to be entirely modality specific (Wallace, Ramachandran, & Stein, 2004). This may explain how a signal in one modality can influence the processing and perception of a signal in another modality. However, as only a few examples have been documented so far, it is not yet clear whether such cross‐modal effects are exploited in courtship displays.

A study on mate choice in Túngara frogs showed that two courtship signals which are not attractive individually, that is the two parts of the vocal signal, become attractive for the females when combined with a visual signal (the inflation of the vocal sac) (Taylor & Ryan, 2013). The multimodal integration of signals by females is therefore not additive but rather involves a complex emergence. In another recent paper on Túngara frogs, Stange et al. (2016) tried to assess the relative importance of each part of a multicomponent signal by manipulating the complexity of the courtship display. They found that female integration of the multicomponent male signal was not additive and that the preference varied with display complexity, that is with the number of components of the signal. This suggests the presence of some higher‐order interaction between the visual and acoustic components of the courtship that goes beyond a simple enhancement effect. Finally, in the brown‐headed cowbird (*Molothrus ater)*, the intensity of the visual component of male courtship modulates the attractiveness of male songs (Ronald et al., 2017). This study is an interesting example where two components of male courtship interact in a complex way to modify overall attractiveness. Examples of this kind are still scarce, but there is no doubt that the growing interest in multimodal signalling displays will allow more research to reveal similar interactions in the courtship displays of other species.

## 4 CONCLUSION

Over the past years, many authors have proposed new theoretical backgrounds for the study of the role and function of multimodal displays (Bro‐Jørgensen, 2010; Candolin, 2003; Partan, 2013; Rowe & Halpin, 2013). Even though courtship displays typically involve signals coming from more than one sensory modality, relatively little theoretical and experimental work exists on multimodal and multicomponent courtship displays, and most of the work so far has focused on auditory or visual courtship. Empirical studies are now needed to specifically test how the variation in different modalities and the interaction between them influence female response and choice in the context of courtship. In addition, we know very little about the neural mechanisms involved in multisensory processing in the courtship context. As mentioned above, uni‐sensory information from different sensory channels is integrated and transformed into multisensory responses in the midbrain (e.g., Gandhi & Katnani, 2011; Meredith & Stein, 1986) and cross‐modal stimuli yield faster responses and can be detected with higher accuracy than modality‐specific stimulus presentations (Gingras, Rowland, & Stein, 2009). Whether these neural and behavioural principles of multi‐sensory enhancement also hold in the context of multimodal courtship displays remains to be investigated.

Finally, courtship is often an interactive process between two sexes rather than the production of signals by a courting individual and its evaluation by a receiver. In a number of dynamic courtship interactions, the emitter modifies its signals on the basis of the response of the receiver. Therefore, many types of multimodal courtship can be fully understood only by analysing their variation across time and in response to signals coming from the receiver. This is a further level of complexity that we have only started to explore.
